# F-box proteins and cancer: an update from functional and regulatory mechanism to therapeutic clinical prospects

**DOI:** 10.7150/thno.42735

**Published:** 2020-03-04

**Authors:** Dinesh Singh Tekcham, Di Chen, Yu Liu, Ting Ling, Yi Zhang, Huan Chen, Wen Wang, Wuxiyar Otkur, Huan Qi, Tian Xia, Xiaolong Liu, Hai-long Piao, Hongxu Liu

**Affiliations:** 1 Department of Thoracic Surgery, Cancer Hospital of China Medical University, Liaoning Cancer Hospital & Institute, Shenyang 110042, China.; 2 CAS Key Laboratory of Separation Science for Analytical Chemistry, Dalian Institute of Chemical Physics, Chinese Academy of Sciences, Dalian 116023, China.

**Keywords:** F-box, E3 ligase, ubiquitin, substrate, cancer progression

## Abstract

E3 ubiquitin ligases play a critical role in cellular mechanisms and cancer progression. F-box protein is the core component of the SKP1-cullin 1-F-box (SCF)-type E3 ubiquitin ligase and directly binds to substrates by various specific domains. According to the specific domains, F-box proteins are further classified into three sub-families: 1) F-box with leucine rich amino acid repeats (FBXL); 2) F-box with WD 40 amino acid repeats (FBXW); 3) F-box only with uncharacterized domains (FBXO). Here, we summarize the substrates of F-box proteins, discuss the important molecular mechanism and emerging role of F-box proteins especially from the perspective of cancer development and progression. These findings will shed new light on malignant tumor progression mechanisms, and suggest the potential role of F-box proteins as cancer biomarkers and therapeutic targets for future cancer treatment.

## Introduction

Ubiquitination is one of the key post translational modification which is regulated by cascade of three component enzymes including ubiquitin activating E1 enzyme, ubiquitin conjugating E2 enzyme and ubiquitin-protein E3 ligase. In the ubiquitination system, ubiquitin (Ubi) is a polypeptide of 76 amino acids in length, it is activated by an E1, delivered to an E2 by the E1, and finally an E3 interacts with the Ubi-loaded E2 and recognizes a specific “motif” at the substrate and links the self-lysine residue to lysine residues at the target protein. Ubiquitylation is initially described as a process that induces substrate degradation and erases the unfavorable products 1. Subsequently, different consequences have also been identified, such as signal transduction, enzyme stabilization or activation. Emerging evidences have revealed that ubiquitination participates in nearly all kinds of biological processes including cell cycle, transcription, and various signaling pathways. Dysregulations of ubiquitination will induce multiple diseases, including neurodegenerative diseases, inflammatory disorders and various types of cancers especially.

**Figure 1:**
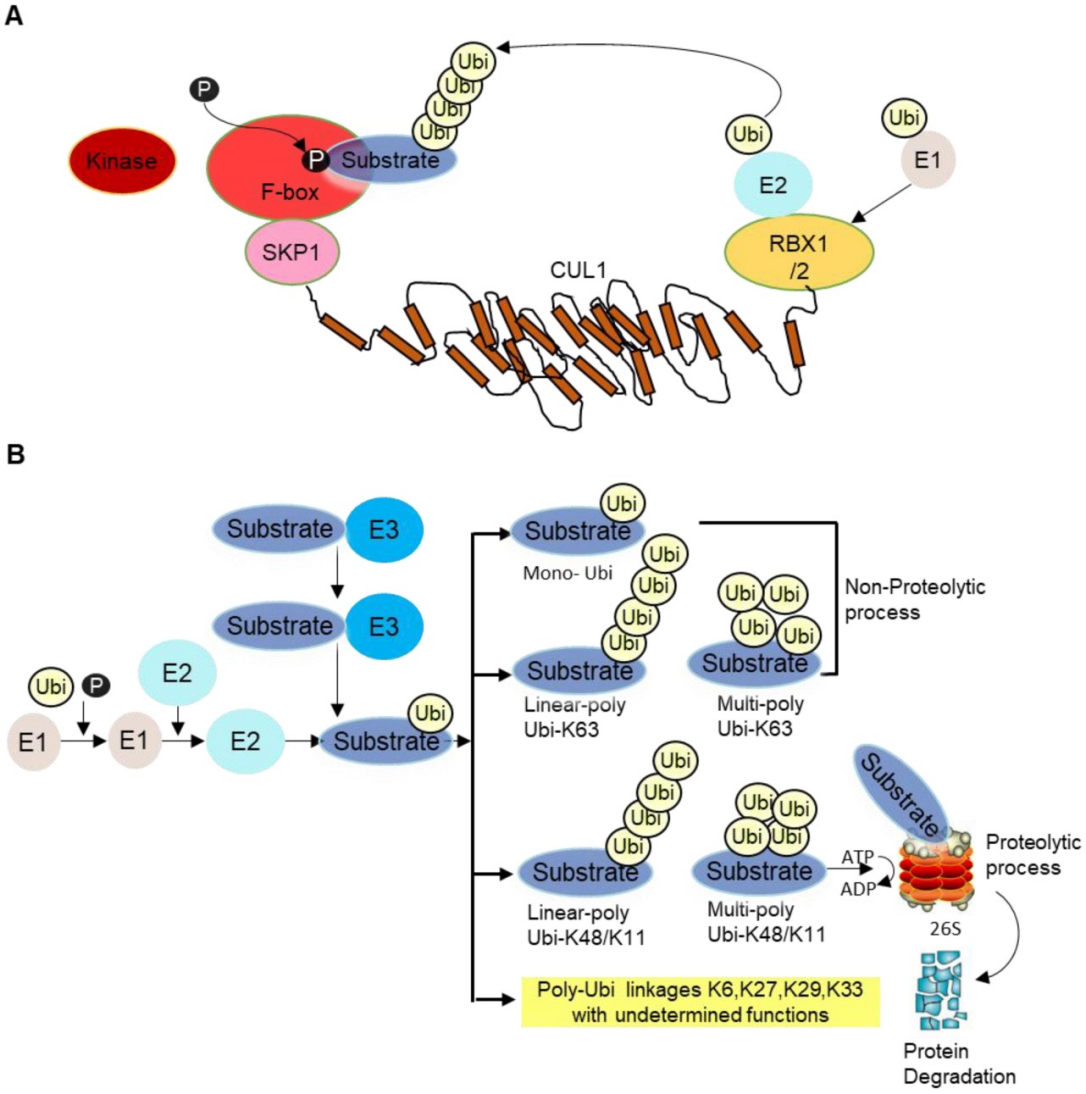
** A** SCF complex. This complex comprises of scaffold CUL1, SKP1, RBX1/2 and F-box receptor. The substrate is phosphorylated by specific kinase enzyme and recognized by the substrate recognition domain. Ubi is transferred from E2 to E3 ligase/F-box proteins in coordination with RBX1/2 for proteosomal degradation. **B** Different forms of ubiquitination. After the substrate is presented to F-box protein, different types of ubiquitination occur depending on the number and types of Ubi/Ubis presented to substrates, namely mono-ubiquitination (i.e., single ubiquitin is added to substrate), linear poly Ubi-K63/K48/K11 (many Ubis are added one after another along a line format at lysine K63/K48/K11 locus of Ubis) and multi-poly-Ubi-K63 (Ubis are added in multilayers at lysine K63/K48/K11 locus). Some other Ubi-types are yet undetermined, such as K6, K27, K29, K33, etc. Ubi-K48/K11 types are proteolytic whereas Ubi-K63 and mono-Ubi are non-proteolytic in nature. The substrate is mainly processed at the 26S proteasome complex for ubiquitination guided proteasomal degradation.

E3 ligase is the core component of the ubiquitination cascade, because they control the substrate specificity and binds to the substrates directly. There are hundreds of E3 ubiquitin ligases in humans 2. Among them, the SKP1-cullin 1(CUL1)-F‑box (SCF) E3 ligase complex, is so far the best-characterized E3 ligase family. The SCF complex consists of four subunits, an adaptor protein SKP1, a RING finger protein RBX1/2, a scaffold protein CUL1, and one variable F‑box protein that recognizes specific substrates 3. CUL1 binds to SKP1 and F-box protein at its N-terminus and to the RING protein RBX1/2 at its C-terminus. F-box protein generally recognizes phosphorylated substrate and presents it for ubiquitination (Figure 1A). Then, different types of ubiquitination will occur depending on number and types of Ubi(s) linked to substrates. Lysine 48 (K48) as well as K11 linked Ubi chains are proteolytic in nature whereas K63-linked and mono-Ubi are non-proteolytic in nature (Figure 1B). Although not all F-box proteins are well-characterized, multiple F-box proteins like SKP2, FBXW7, FBXO4, FBXO32 have been linked to cancer development, progression as well as cancer cachexia 4.

Here, we will discuss the molecular regulatory mechanisms, especially those involved in cancers, for F-box proteins, summarize the F-box protein relevant small compound inhibitors, and envision the future perspectives of F-box protein targeted cancer treatment.

## Classification of F-box proteins

F-box proteins are broadly classified into three sub-families: i) FBXL: F-box with leucine rich amino acid repeats, ii) FBXW: F-box with WD 40 amino acid repeats, iii) FBXO: F-box only with uncharacterized domains. There are about 22 FBXLs, 10 FBXWs and 37 FBXOs in the human genome at present.

## Substrate recognition mechanism

F-box proteins generally recognize substrates modified by proper post-translational modification, especially phosphorylation. For example, substrates of FBXW7 contain the conserved CDC4 phospho-degron sequence 4-X‑pThr (or pSer)‑Pro‑Pro‑X‑pSer (or pThr, Glu or Asp) (X represents any amino acid) 5, 6. Only when these amino acids get phosphorylated, FBXW7 can recognize and ubiquitinate the substrates for degradation. In some other cases, dephosphorylated degrons can also be recognized and ubiquitinated. When phospho-Tyr-655 is dephosphorylated by protein Tyr phosphatase L1 (PTPL1), p85β binds to FBXL2 and gets ubiquitinated 7. In addition, the substrates can also be modified by glycosylation or mannose oligosaccharide 5, 6, 8, 9. For instance, FBXO6 ubiquitinates the glycosylated degron in T-cell receptor alpha chain 9, and FBXO2 ubiquitinates N‑linked high-mannose oligosaccharides of precursor β1 integrin 8 of the respective substrates. Very rarely, the substrates are modified by lysine acetylation or tyrosine phosphorylation. Of note, the ubiquitination process can be nullified (or deubiquitinated) by deubiquitinating enzymes with evidences in various types of mammalian cell systems 10.

## F-box proteins are involved in multiple cancer hallmark pathways

Biological functions of F-box proteins are well characterized in cells, mouse models and human cancer tissues. Many F-box proteins are found to act as either direct tumor suppressors/oncogenes or indirect cancer regulators. Their key functions largely depend on their ubiquitination abilities on substrates involved in cancer hallmark pathways, including cell cycle, DNA damage, epithelial-mesenchymal transition (EMT) as well as multiple signaling pathways like AKT/PI3K, BMP, p53, NRF2, AMPK/mTOR, AKT, NF-κB and Hippo pathway 7, 11-21 all of which can contribute to tumor growth, proliferation, progression, metastasis and invasion. Functions of F-box proteins and the corresponding substrates are being described briefly in Table 1. In the following parts, we will introduce each individual F-box protein with respect to their molecular function and potential clinical utility in cancer.

## FBXL family

### SKP2

**Figure 2:**
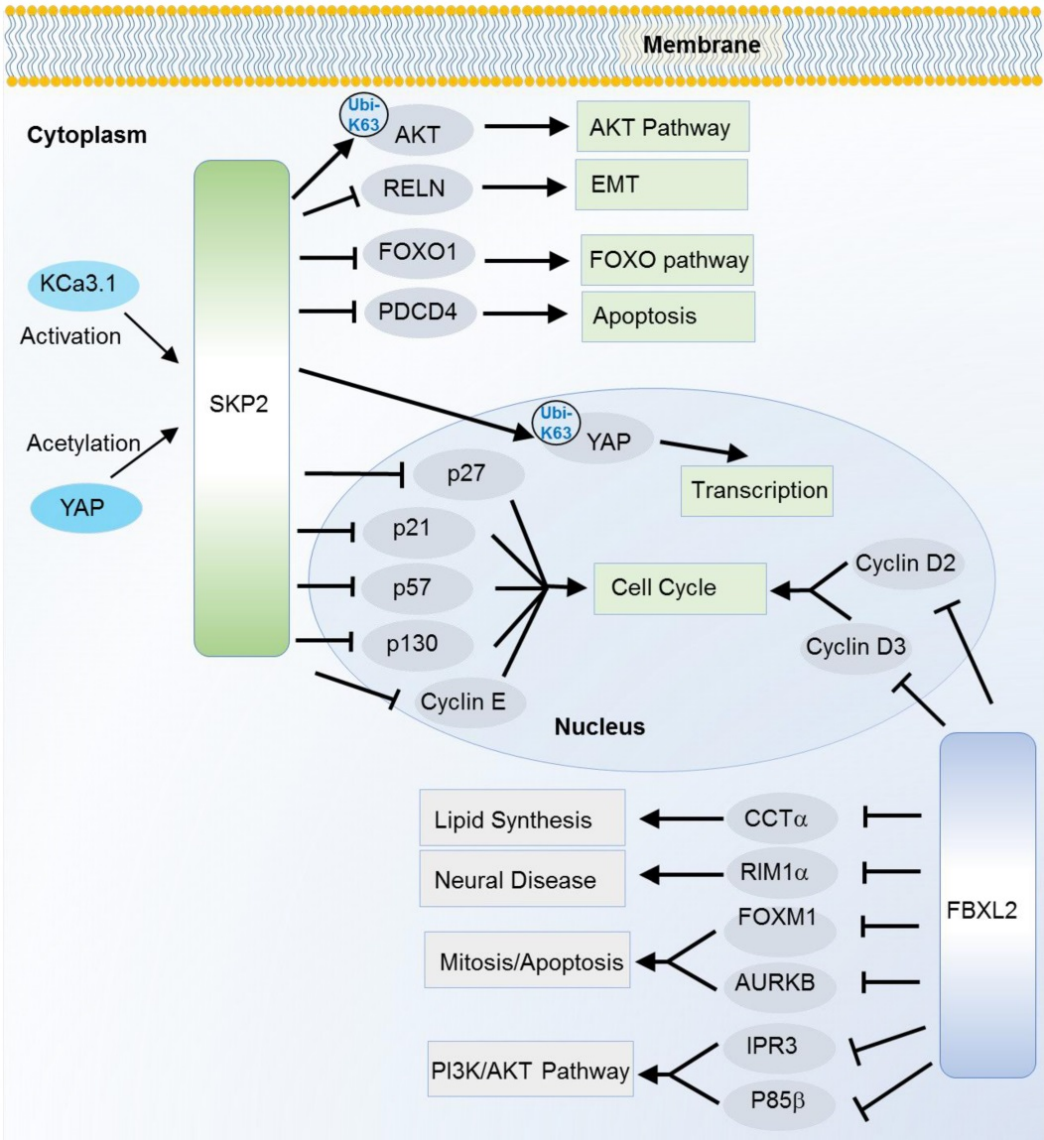
Substrates of SKP2 and FBXL2 in various cancer relevant cellular functions and pathways. K63-linkage poly-ubiquitinations are annotated on the arrows, the others are K48-linkage ubiquitinations.

SKP2, also known as FBXL1, is the most characterized oncogene among F-box members. It is especially well known as a cell cycle regulator that can induce degradation of various cell cycle regulators. Of them, p27, a cyclin-dependent kinase (CDK) inhibitor is one of the best known substrates since the first discovery in 1999 22. Later on, a collection of CDK inhibitors including p21 23, p27 22 and p57 24, and other cell cycle regulators like cyclin E 25, c-Myc 26 and p130 27 are successively identified as the SKP2 substrates. Interestingly, SKP2 not only induces degradation of c-Myc, but also activates c-Myc target genes as a transcription cofactor 26. Recently, a potential up-stream regulator of SKP2 in cell cycle is also revealed. The intermediate conductance calmodulin/calcium-activated potassium channel (KCa3.1) activates SKP2 and promotes cell proliferation, invasion and metastasis by degrading p21 and p27 through SKP2 16. In addition to the cell cycle pathway, SKP2 also participates in other cancer hallmark pathways such as FOXO, AMPK/mTOR, AKT, apoptosis and Hippo signaling pathway 15-18, 20, 21, 28-32 (Figure 2). SKP2 can ubiquitinate and induce the degradation of the transcription factor FOXO1 which possesses a tumor suppressor function 33. SKP2 can regulate the AMPK pathway via degradation of coactivator-associated arginine methyltransferase 1 (CARM1), a member of protein arginine methyltransferase 18. In response to DNA damage, SKP2 enhances phosphorylation and ubiquitination of programmed cell death protein 4 (PDCD4) to inhibit apoptosis with subsequent increase in cell growth and proliferation of breast cancer cells 34. In addition to the degradation effect (mainly mediated by K48 linkage Ubis), SKP2 can also regulated the non-degradation polyubiquitination (mainly mediated by K63 linkage Ubis). The Hippo signaling key factor YAP was stabilized by SKP2 through the K63-linkage ubiquitination 35. Recently, SKP2 has also been found to promote the K63-linkage mediated ubiquitination and activation of AKT, a key factor that conveys growth factor signals from cell outside to inside 36.

Probably because of its proteolysis effects on its substrates like p21, p27, PDCD4 and FOXO1, a large part of which are tumor suppressors, SKP2 mainly play oncogenic functions. Correspondingly, SKP2 up-regulation has been observed in many cancers like malignant oral cancer 37, colorectal cancer 38, hepatocellular carcinoma 18, and breast cancer 34. Besides, the high expression of SKP2 has been implicated as a poor prognosis indicator in several types of cancers 39-41.

### FBXL2

FBXL2 is also known for its active role in cell cycle. However, the ubiquitination substrates are unknown until 2012 when cyclin D2, D3 and Aurora kinase B (AURKB) were successively recognized as the substrates 42-44. Meanwhile, unlike many F-box proteins, FBXL2 does not always recognize the phosphodegron of its substrates. For cyclin D2 and D3, FBXL2 target on their calmodulin-binding motifs. As mentioned above, FBXL2 also regulates PI3K/AKT pathway through targeting and degrading the dephosphorylated p85β (a catalytic subunit of PI3K pathway) 7. Besides, FBXL2 can ubiquitinate phosphorylated forkhead box M1 (FOXM1), a transcriptional factor which regulates the expression of several cell cycle genes including cyclin B1 and D1 45 (Figure 2). Due to its ubiquitin mediated degradation of cell cycle activators like cyclin D2, cyclin D3 and FOXM1, the FBXL2 mainly play a tumor suppressive role in several types of cancers like gastric cancer and leukemia 42, 43, 45. However, its tumor suppressive role is controversial. In a recent study, inhibition of FBXL2 may also promote apoptosis and limit tumor growth in PTEN-null cancers where PTEN has been identified as a counteractor of FBXL2 in binding with IP3R3 (a major player in Ca+ dependent apoptosis) for ubiquitin mediated degradation 11.

### FBXL3

FBXL3 mainly participates in mammalian oscillating circadian clock system through ubiquitinating two inhibitors of CLOCK-BMAL1 complex, cryptochrome-1 (CRY1) and cryptochrome-2 (CRY2), since the simultaneous discovery by several research groups in 2007 46-49. FBXL3 mediated degradation of CRY1 and CRY2 can re-activate the CLOCK-BMAL1 complex and increase the protein levels of period circadian protein homolog 1 (PER1) and 2 (PER2), two circadian clock regulators with tumor suppressor activity 49. Nearly ten years later, another unexpected regulatory function between FBXL3 and CRY2 is found that FBXL3 in cooperation with CRY2 targets an oncogenic substrate c-Myc to inhibit uncontrolled tumor cell growth 50. Recently, phosphorylated Tousled-like kinase (TLK2) is also found to be ubiquitinated by FBXL3 in cooperation with CRY2 in a cell cycle regulating mechanism 51. Thus, FBXL3 is a remarkable molecular connection between circadian and cell cycle, and with tumor suppressive potentials.

### FBXL4

There are two revealed substrates, lysine demethylase 4A (KDM4A, also called JMJD2A) and GABA_A_ receptor resistant to dieldrin 10, for FBXL4 at present. FBXL4 was firstly found to regulate replication time by degrading the substrate KDM4A in 2011 52. Later on, it was also revealed to regulate the timing of sleep through ubiquitin mediated degradation of RDL 53. Despite the limited knowledge about its substrates, it is a potential tumor suppressor. Loss of FBXL4 gene is associated with advanced tumor stage and poor survival in prostate cancer 54. Detection of deleted variants of FBXL4 in circulating tumor cells suggests it as a potential prognostic biomarker 54. FBXL4 is also associated with mitochondrial DNA depletion syndrome and intellectual disabilities 55.

### FBXL5

**Figure 3:**
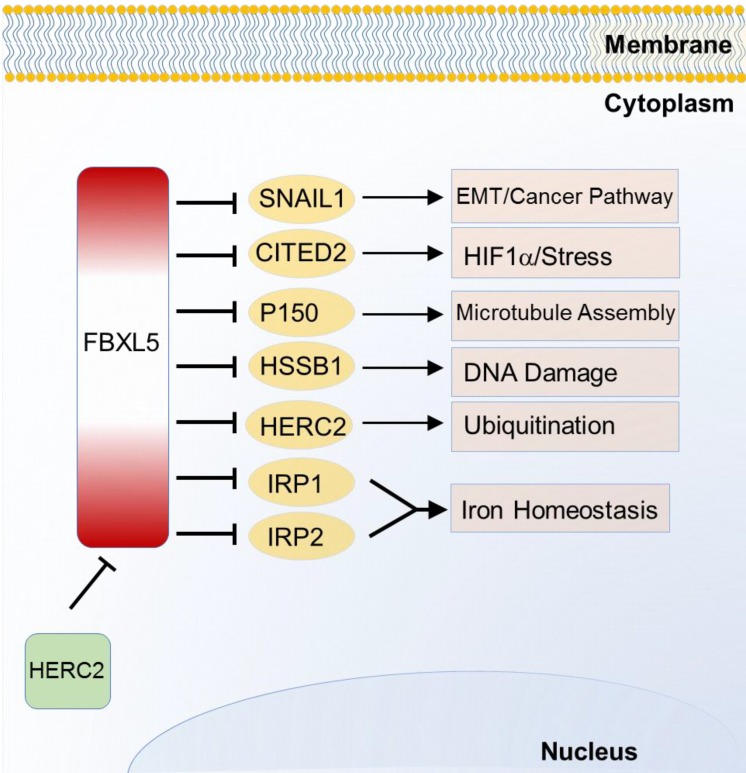
Substrates of FBXL5 in iron homeostasis and various cancer relevant cellular functions and pathways.

FBXL5 is the first SCF E3 ligase identified to regulate homeostasis or iron metabolism. Iron regulatory protein 1 (IRP1) and 2 (IRP2), two post transcriptional regulatory genes, can control and maintain cellular iron uptake, use, release and storage. FBXL5 was found to target and degrade IRP1 and IRP2 through ubiquitination in 2009 56. Unlike other F-box protein members, FBXL5 possesses an iron and oxygen binding hemerythrin domain that acts as a specific motif-dependent regulator for FBXL5-self differential stability 56. Self-renewal of hematopoietic stem cell without FBXL5 can no longer survive due to cellular iron overload 57. Recently, FBXL5 is found to be ubiquitinated by HECT and RLD domain containing E3 ubiquitin protein ligase 2 (HERC2) for proteasomal destruction. When FBXL5-HERC2 interaction is blocked, stability and abundance of FBXL5 is increased with lower intracellular Fe^2+^ load 58. Emerging evidences of FBXL5-IRP2 axis suggest its potential therapeutic implication in cancer and hematopoietic stem cells 56, 57. Additionally, FBXL5 also triggers chromosomal instability by degrading p150 which is required for binding to dynein and microtubules 59. Recent studies also find that FBXL5 targets on Snail homolog 1 (SNAIL1) 60, Cbp/p300-interacting transactivator 2 (CITED2) 61 and human single-strand DNA binding proteins 1 (HSSB1) 62 which are respectively involved in EMT, HIF signaling pathway and DNA damage response (Figure 3).

### FBXL7

The most well-known substrate of FBXL7 is Aurora A kinase (AURKA) 63, a pivotal regulator of mitosis. Interestingly, the ubiquitination between FBXL7 and AURKA only occurs during mitosis although FBXL7 co-localizes with AURKA throughout cell cycle 63. FBXL7 can also regulate mitochondrial function by ubiquitinating survivin for degradation 64. Interestingly, AURKA restricts the ubiquitination of survivin by tightly regulating FBXL7, thereby promoting gastric cancer resistance to drug 65. Transcript level of FBXL7 is very high and associated with poor prognosis and unfavorable response to paclitaxel-based chemotherapy in ovarian cancer patients 66. In addition, FBXL7 is a target of FBXL18 for polyubiquitination and proteasomal degradation to regulate the cell cycle progression 67.

### FBXL12

FBXL12 also regulates cell cycle. It can induce calcium/calmodulin-dependent protein kinase (CaMKI) polyubiquitination guided proteasomal degradation to attenuate p27 phosphorylation and disrupt cyclin D1/CDK4 complex assembly and G1 arrest in lung epithelia 68. FBXL12 can also augment p21 by mixed-type ubiquitination, including both K48 and K63 linked Ubi chains 69. On the other hand, FBXL12 is mostly distributed in thymus and regulates the T-cell differentiation. FBXL12 regulates transition or T cell differentiation from CD4^+^CD8^+^ cells into CD4^-^CD8^+^/CD4^+^CD8^-^ cells through degradation of aldehyde dehydrogenase 3 (ALDH3). The level of FBXL12 diminishes as T cells (CD4^+^CD8^+^ cells) progress into CD4^-^CD8^+^/CD4^+^CD8^-^ cells, suggesting the key role of FBXL12-ALDH3 axis in the maturation of undifferentiated thymocytes 70. Besides, FBXL12 ubiquitinates and degrades one sub-unit of the Ku heterodimer, Ku80, a key regulator for the nonhomologous end joining double strand break repair pathway 71.

### FBXL13

FBXL13 is abundant at centrosome and is associated with chromosomal stability. It interacts with centrosome associated proteins Centrin-2, Centrin-3, CEP152 and CEP192 72. Of these proteins, accumulation of CEP192 isoform is harmful to cells by increasing centrosome over-duplication that can promote cancer cell invasion and metastasis. FBXL13 targets CEP192 for proteasomal degradation to lower centrosomal γ-tubulin and disrupt microtubule array formation.

### FBXL14

FBXL14 mainly regulates the EMT pathway by degradation of the EMT inducers SNAIL1 73 and Twist-related protein 1 (TWIST1) 74. In pancreatic cancer, liver kinase B1 (LKB1) promotes the ubiquitination of SNAIL1 by FBXL14, suggesting LKB1/FBXL14/SNAIL1 axes a potential therapeutic target 75. One key oncogene c-Myc is also ubiquitinated by FBXL14 for proteasomal degradation, and this ubiquitination can be reversed back by a deubiquitinase USP13 in glioma stem cells. The antagonistic relation between USP13 and FBXL14 deserves deep studies for further clinical and therapeutic applications 76. Additionally, FBXL14 targets and degrades CUB domain-containing protein 1 (CDCP1) to reduce its stability and prevent CDCP1 target genes involved in breast cancer metastasis 77. FBXL14 even reaches neuronal differentiation by targeting C-terminal WRPW motif in a Notch signaling factor, hairy and enhancer of split 1 (HES1) 78.

### FBXL17

FBXL17 degrades suppressor of fused homolog (SUFU) to release glioma-associated oncogene (GLI) from the SUFU domain for proper Hedgehog signaling pathway. Lack of FBXL17 often causes defective Hedgehog signaling, a characteristic of impaired cancer cell proliferation and medulloblastoma tumor growth 79. FBXL17 is also a regulator of NRF2 oxidative stress pathway by degradation of transcription regulator protein BACH1 14. In addition, FBXL17 is a quality control factor for dimeric BTB complexes 80.

### FBXL18

As mentioned above, FBXL18 can mediate the ubiquitination and degradation of FBXL7, thus indirectly impact cell cycle progression 67. In another study, FBXL18 inhibits apoptosis and exerts an oncogenic function through K63-linked ubiquitination of AKT in glioma 15. Recently, FBXL18 is found to ubiquitinate xeroderma pigmentosum group B complementing protein (XPB) where the CDK7 triggers Ser90 phosphorylation of XPB and presents XPB to FBXL18 81.

### FBXL19

FBXL19 is one F-box protein showing self-induced ubiquitination. An acetyltransferase CBP catalyzes acetylation of FBXL19. Stability of FBXL19 is increased with the level of CBP and vice versa 82. Additionally, FBXL19 targets lysine-166 of Rac family small GTPase 3 (RAC3) for proteasomal degradation to regulate TGFβ1-induced E-cadherin down-regulation in esophageal cancer cells 83. In lung epithelial cells, FBXL19 induces ubiquitination and degradation of ras homolog family member A (RhoA) by binding cytoplasmic small GTPase at lysine-135 of RhoA. Consequently, phosphorylation of p27 and cell proliferation is reduced. Of note, phosphorylation of RhoA is mediated by protein kinase ERK2. Thus, FBXL19 regulates the cell proliferation and cytoskeleton rearrangement 84.

### The other FBXLs

Although FBXL is the most comprehensively described F-box family protein, there are still several FBXLs with only few substrates. FBXL10 and FBXL11, although contain the FBXL domains, are better known as two histone demethylases KDM2B and KDM2A. Their ubiquitination substrates are still unclear. FBXL6, FBXL8, FBXL9 and FBXL16 are orphan E3s without any known substrates at present. Our previous computational study predicts some substrates for the FBXLs including the orphan ones, e.g., voltage-dependent anion-selective channel protein 2 (VDAC2) and cyclin-A2 are predicted as substrates of FBXL6 85. FBXL15 can regulate BMP signaling pathway by degradating its ubiquitinated substrate SMAD ubiquitination regulatory factor 1 (SMURF1) 12. FBXL20 can ubiquitinate Vacuolar protein-sorting 34 (Vps34), a regulator involved in autophagy and receptor degradation 86 , and E-cadherin for degradation 87. Like FBXL3, FBXL21 also targets on both CRY1 and CRY2 for degradation, but its interaction mainly occurs in the cytoplasm, and it antagonizes the degradation induced by FBXL3 in the nucleus 88.

## FBXW family

### β-TrCP (FBXW1 or FBXW11)

β-TrCP has two main isoforms β-TrCP1 (also called FBXW1) and β-TrCP2 (also called FBXW11) 89. Although specific substrates of different isoforms are observed, most of the substrates are common between the two main isoforms. The functional specificity of these isoforms is yet to be elucidated. Here, we shall use β-TrCP to refer to both of them, or otherwise specified.

**Figure 4:**
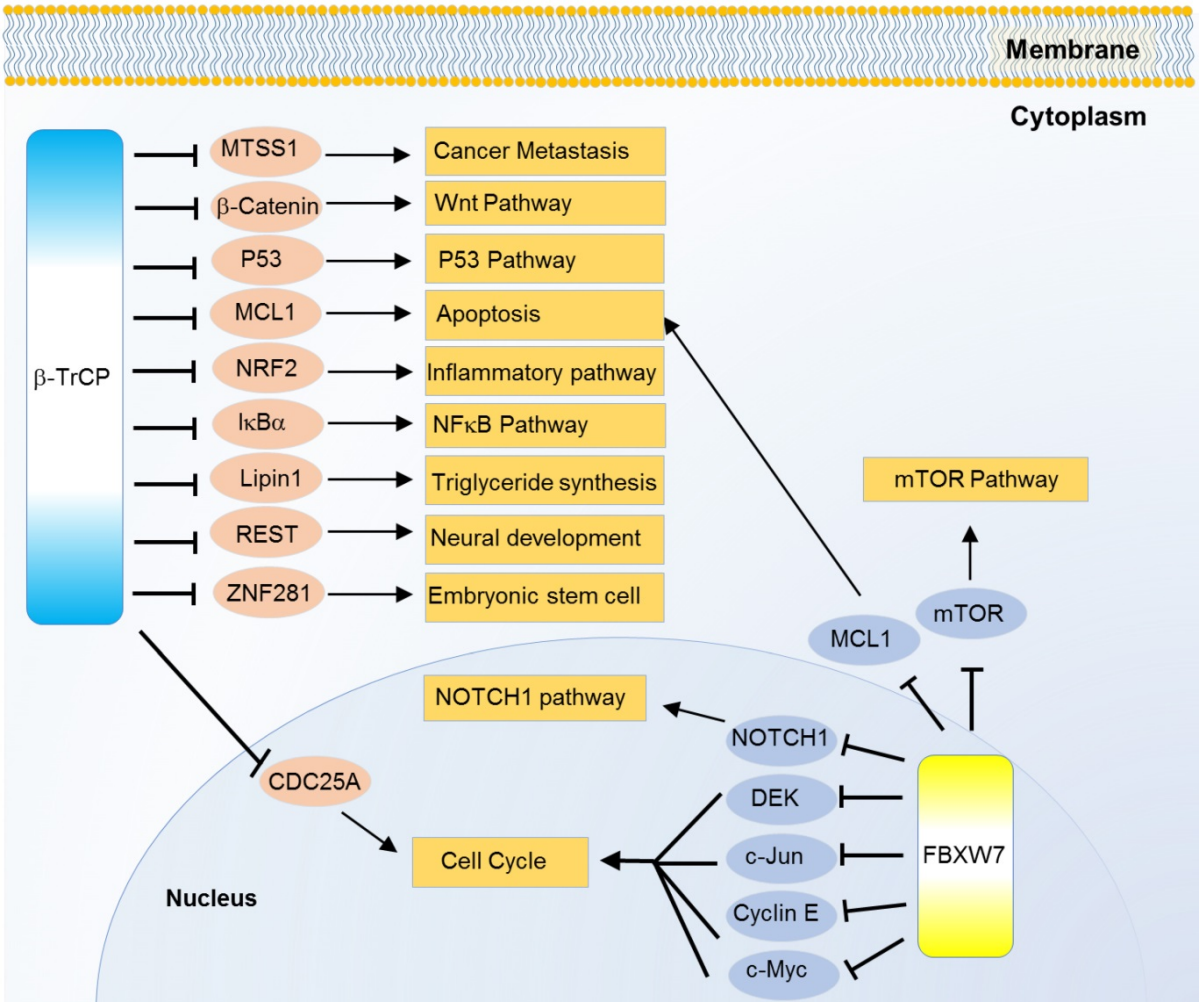
Substrates of FBXW family proteins β-TrCP and FBXW7 in various cancer relevant cellular functions and pathways.

Way back to 1990s, β-TrCP was found as a regulator of β-catenin and was one main regulator of cell viability. β-TrCP recognizes the Ser33 and Ser37 of β-catenin phosphorylated by glycogen synthase kinase 3β (GSK3β) 90, 91. It also interacts with the phosphorylated domains in IκBα and mediates IκBα ubiquitination, thus activating the NF-κB pathway 92, 93. When DNA damage or stalled DNA replication occurs, the activated checkpoint kinase-1 (CHK1) and 2 (CHK2) trigger hyperphosphorylation of cell division cycle 25A (CDC25A), then β-TrCP targets CDC25A for ubiquitin-mediated proteolysis, and delays the cell cycle progression. Thus, β-TrCP regulates normal cell cycle progression and acts like cell cycle check-points 94. Oncogenic transformation and neural differentiation are also controlled by β-TrCP through targeting and ubiquitinating RE1-silencing transcription factor (REST). Over-expression of β-TrCP is commonly found in cancers with low level of REST 95. High level or truncated REST in cancer cells causes genomic instability which leads to oncogenic cellular transformation. In few cases, β-TrCP also acts like a tumor suppressor gene. The SCF-β-TrCP dependent ubiquitination guided degradation of REST during G2 phase increases the optimum time for activation of spindle check points 96. β-TrCP also stimulates GSK3β mediated apoptosis where GSK3β phosphorylates the protein, induced myeloid leukemia cell differentiation protein Mcl-1 (MCL1), and the MCL1 is then recognized by β-TrCP for proteasomal degradation 97. Another substrate of β-TrCP is NRF2 which is also GSK3β dependent 98. β-TrCP with the UbcH5 ubiquitin-conjugating enzyme which helps to form heterotypic polyubiquitin chains on c-Myc can induce ubiquitination mediated stabilization of c-Myc 99. β-TrCP degrades phosphorylated LPIN1, a factor of fatty acid biosynthesis. Thus, the role of β-TrCP becomes clear in lipid metabolic homeostasis 100 (Figure 4).

Some isoforms specific mechanisms are also revealed. β-TrCP, especially β-TrCP1, and IκB kinase 2 (IKKβ) can, in part, regulate the loss of function of p53. In this case, IKKβ phosphorylates p53 at ser362 and ser366 positions, then β-TrCP1 recruits the phosphorylated p53 for ubiquitination guided degradation 101. Besides, β-TrCP1 promotes another F-box protein FBXW2 ubiquitination, and so does FBXW2 to SKP2, then the β-TrCP1-FBXW2-SKP2 axis presents an oncogene-tumor suppressor-oncogene cascade that controls cancer cell growth 102. β-TrCP2 but not β-TrCP1 can mediate the ubiquitination and degradation of ZNF281, thus inhibiting the progression of colorectal cancer 103. In addition, mainly β-TrCP1, can ubiquitinate and degrade MTSS1 in prostate and breast cancers 104 (Figure 4). Notably, β-TrCP1 and β-TrCP2 are observed to target each other for degradation in a recent study and β-TrCP2 preferentially degrades DEPTOR and REDD1, two inhibitors of mTORC1, thereby inhibiting autophagy and promoting cell growth 105.

### FBXW7

FBXW7 (also known as hCdc4, SEL10) is another well characterized member of F-box family with WD40 repeat. There are three FBXW7 isoforms, FBXW7α, FBXW7β, and FBXW7γ in mammalian cells. These isoforms have different cellular localizations: FBXW7α is localized in the nucleoplasm, FBXW7β is in cytoplasm, and FBXW7γ is nucleolar. FBXW7α is most ubiquitously one and performs most of the recognized functions. Here, we mainly use FBXW7 to represent FBXWα. Since the first identified oncogenic substrate cyclin E 106, FBXW7 is recognized to ubiquitinate multiple oncogenic substrates like NOTCH1 107, JUN 108, c-Myc 109, mTOR 110, MCL1 111 and DEK 112 for degradation (Figure 4). Meanwhile, mutation mediated down regulation of FBXW7 is common in various types of cancers, especially T cell acute lymphatic leukemia and cholangiocarcinoma 113. Notably, FBXW7 mutation reduces the binding affinity to NOTCH and *knocked-out* FBXW7 increases the level of NOTCH1-NICD, c-Myc as well as HIF-1α activity in chronic lymphoid leukemia 114. Growth and progression of cholangiocarcinoma cells can also be regulated by FBXW7. FBXW7, in some aspects, is a p53 dependent tumor suppressor gene. In a search to rule out the relation between FBXW7 and p53, several putative DNA response elements were respectively identified at the FBXW7α, FBXW7β and FBXW7γ isoforms. siRNA *knocked-down* FBXW7 MEF (under p53^+/-^ condition) cells show growth advantage than controls, and p53^-/-^ MEF cells show similar growth like controls 115.

### The other FBXWs

Some of the other FBXWs also participate in cancer relevant processes. FBXW2 can target on SKP2 for degradation, thus stabilizing the substrates of SKP2 102. FBXW2 also ubiquitinates β-catenin for degradation 116. FBXW5 mediates the ubiquitination and subsequent degradation of a tumor suppressor TSC2 117. FBXW5 also regulates cell cycle by ubiquitination and subsequent proteasomal degradation of spindle assembly abnormal protein 6 (SASS6) 118 and epidermal growth factor receptor kinase substrate 8 (EPS8) 119 during S and G2 phase respectively. FBXW8 can ubiquitinate and degrade MAP4K1, thereby affecting cell proliferation and differentiation 120. Substrates of the other FBXWs remain to be explored.

## FBXO family

### FBXO1

**Figure 5:**
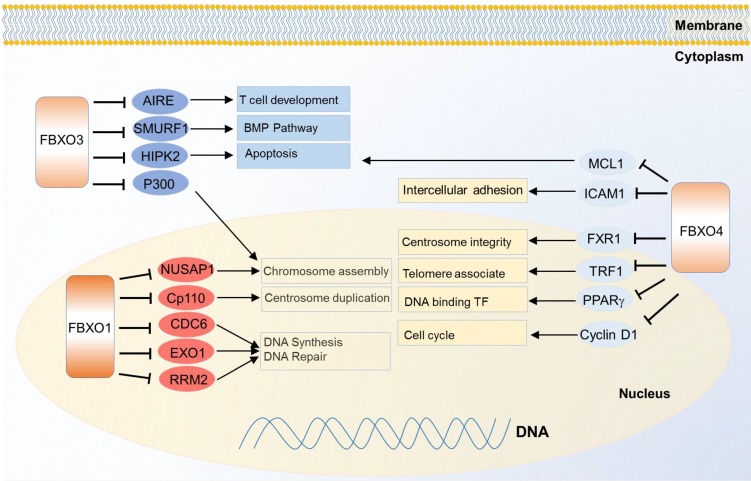
Substrates of FBXO family proteins FBXO1, FBXO3 and FBXO4 in various cancer relevant cellular functions and pathways.

FBXO1 is also called cyclin F because it contains a cyclin box domain, however, it also functions through SCF E3 ligase complex. It mainly localizes in the nucleus, and participates in centrosome duplication and DNA repair. The first identified substrate of FBXO1 is centriolar coiled-coil protein of 110 kDa (CP110) which is necessary for centrosome duplication, and the FBXO1 mediates degradation of CP110 121. It also controls the maintenance of genome stability by degradation of ribonucleoside-diphosphate reductase subunit M2 (RRM2), which converts ribonucleotide to deoxyribonucleotide required for DNA replication and DNA repair 122. Nucleolar and spindle-associated protein 1 (NUSAP1), a cell-cycle-regulated microtubule-binding protein involved in chromosome assembly, is one substrate of FBXO1 as well 123. In another case, FBXO1 degrades eukaryotic DNA replication protein CDC6 and blocks DNA replication at the end of mitosis, thus inhibiting the progress of error DNA synthesis to attain genomic stability 124 (Figure 5). Meanwhile, down regulation of FBXO1 is associated with advanced tumor stage, poor survival and accelerated tumor growth in hepatocellular carcinoma 125.

### FBXO3

FBXO3 may generate effects on cancer cells through multiple pathways. It was identified to regulate apoptosis by degradation of two transcription factor co-activators homeodomain-interacting protein kinase 2 (HIPK2) and p300. Meanwhile, protein PML protects them from the degradation without influencing on their ubiquitinations, thus PML, HIPK2 and FBXO3 cooperatively activating p53 dependent transactivation 126. FBXO3 also participates in immune and inflammatory regulation. It stimulates cytokine secretion from human inflammatory cells by destabilizing the phosphorylating FBXL2, a TRAF inhibitor. TRAF is generally involved in responses ranging from tissue injury to cytokine release 127. FBXO3 regulates T cell development by degrading autoimmune regulator (AIRE) which helps eliminate auto-reactive T cells during development, and it increases the AIRE's binding affinity to the positive transcription elongation factor b (P-TEFb) to properly monitor the transcription and directs proper expression of AIRE-responsive tissue-specific antigens in the thymus 128. Besides, FBXO3 can regulate BMP signaling through ubiquitination guided degradation of SMURF1 129.

### FBXO4

FBXO4 also plays important functions in cancer. One of the best known substrates is cyclin D1 since the discovery in 2006. FBXO4 promotes ubiquitin-mediated degradation of Thr286-phosphorylated cyclin D1 130. Correspondingly, FBXO4 dysfunction can contribute to cyclin D1 overexpression and promote malignance in a large fraction of human cancers like melanoma 131 and esophageal cancer 132. Another critical substrate of FBXO4 is telomeric repeat binding factor 1 (TRF1), a negative regulator of telomere length. FBXO4 regulates the ubiquitin-dependent degradation of TRF1 through an atypical small GTPase domain, thereby promoting telomere elongation 133. In two recent studies, there emerges a feedback loop mechanism to balance the level of both FBXO4 and one of its substrate, fragile X mental retardation syndrome-related protein 1 (FXR1), in both head and neck squamous cell carcinoma and prostate cancer 134, 135. FBXO4 also shows tumor suppressive functions in breast cancer and lung cancer through ubiquitin dependent degradation of intercellular adhesion molecule 1 (ICAM1) 136 and MCL1 137 respectively. Besides, FBXO4 mediates ubiquitination of peroxisome proliferator-activated receptor gamma (PPARγ) with cooperation of heat shock 20 kDa-like protein p20 (HSP20) 138 (Figure 5).

### FBXO6

FBXO6 regulates CHK1 ubiquitination and degradation and this may influence drug sensitivity to cisplatin 139, 140. FBXO6 can also control the endoplasmic reticulum stress induced apoptosis by targeting endoplasmic oxidoreductin-1-like protein (Ero1L) for degradation 141. Recently, an evidence has emerged that FBXO6 regulates the genomic stability via chromosome arrangement by monitoring two substrates, mitotic arrest deficient 2-like protein 1 (MAD2) and BUB1-related protein 1 (BUBR1) 142.

### FBXO7

FBXO7 has shown oncogenic potentials 143. However, different from most F-box proteins, most of FBXO7's interacting proteins are not its ubiquitination substrates. For instance, FBXO7 interacts with CDK6, and the interaction is necessary to regulate entry of cell cycle. In a nude mouse experiment, over-expression of FBXO7 transformed the murine fibroblast into tumorigenic cells in a CDK6 dependent manner 144. In another study, *knock- down* of FBXO7 increases the cell proliferation with reduced cell size and mitotic time 145. Besides, over-expressing of FBXO7 increases the development of T cell lymphoma in p53 null cells. Thus, FBXO7 negatively regulates the proliferation and differentiation in a p53 dependent manner 143. Although most of the above oncogenic functions are independent of ubiquitination, some cancer relevant substrates are also revealed. For example, FBXO7 can control apoptosis by ubiquitination guided degradation of human inhibitor of apoptosis protein 1 (cIAP1) whose function is increasingly involved in cell cycle and signaling pathway 146.

### FBXO11

FBXO11 is most likely a tumor suppressor. It can target on a collection of oncogenic substrates. For instance, B-cell lymphoma 6 protein (BCL6), the product of a proto-oncogene, is ubiquitinated and degraded by FBXO11 and the *FBXO11* gene is frequently deleted or mutated in diffuse large B-cell lymphomas 147. EMT and metastasis factor of SNAIL1 and critical oncogenic protein HIF-1α can also ubiquitinated by FBXO11 for degradation 148, 149. In addition, FBXO11 is also known as neddylating E3 ligase, which covalently conjugates NEDD8 to its substrates. One neddylation substrate is p53, FBXO11 can inactivates p53 by inhibiting its nuclear translocation 150. Moreover, a lower expression of FBXO11 implicated poor prognosis in cancer patients 151.

### FBXO31

FBXO31 may also act as a tumor suppressor. It is encoded in the 16q24.3 region in which the loss of heterozygosity is observed in various cancers like breast, ovarian, hepatocellular and prostate cancers. The current identified substrates of FBXO31 include cyclin D1 152, SNAIL 153, MDM2 (an E3 ligase of p53) 154 and mitogen-activated protein kinase kinase 6 (MKK6) 155, all of which are oncogenic or promotive to tumorigenesis. FBXO31 mediates ubiquitination and degradation of these substrates and thereby inhibiting cancer cell development and progression.

### FBXO32

FBXO32 may regulate cancer relevant processes through several targets. It degrades Krueppel-like factor 4 (KLF4) to suppress the breast cancer progression via p38 mitogen-activated protein kinase pathway 156. Like β-TRCP, FBXO32 also degrades IκBα to activate NF-κB, and this degradation happens even under genotoxic and inflammatory stress 157. In another study, FBXO32 ubiquitinates and degrades C-terminal-binding protein 1 (CTBP1), thereby controlling EMT activation 158. Besides, activation of FBXO32 (also known as Atrogin-1) has been linked to cancer induced cachexia 159.

### FBXO45

Lastly, we will update briefly about the role of FBXO45 in cancer. Low level of FBXO45 in gastric cancer tissues was found to be associated with the low survival rate of gastric cancer patients regardless of lymph node metastasis 160. FBXO45 also degrades prostate apoptosis response protein 4 (PAR4) to block selective cell death and promotes cancer cell proliferation and survival 161. FBXO45 can target p73 *in vitro* and *in vivo* to regulate the apoptosis mediated by p53 162. Besides, FBXO45 is also involved in neural development by degradation of the substrate N-Cadherin 163.

### The other FBXOs

Substrates of the other FBXOs are less described, and only limited substrates or interactors are revealed. FBXO5, better knew as early mitotic inhibitor 1 (EMI1), can function as both substrate and inhibitor of the anaphase-promoting complex (APC/CCDH1), which is also an E3 ligase, to start the cell cycle. FBXO10 mediates ubiquitination and degradation of an antiapoptotic protein BCL2. FBXO21 mediates the ubiquitylation and proteasomal degradation of EP300-interacting inhibitor of differentiation 1 (EID1) 164. FBXO22 mediates the ubiquitin-dependent degradation of key sarcomeric proteins, such as alpha-actinin (ACTN2) and filamin-C (FLNC) 165. Notably, FBXO38 mediates the ubiquitination and degradation of the substrate programmed cell death protein 1 (PD-1), a promising cancer immunotherapy target, thus regulating T-cells-mediated immunity 166. Many FBXO members like FBXO15, FBXO16, FBXO20 (also known as LMO7), FBXO24, etc., are still orphan E3 ligases, none of their substrates are characterized.

## Micro-RNAs regulating F-box proteins

Micro-RNAs (mi-RNAs) refer to a class of small regulatory RNAs that control the multiple signaling factors including E3 ubiquitin enzyme of F-box proteins. Here, we have discussed some miRNAs which regulate F-box proteins. The miR-203 promotes cell cycle exit and long term cell proliferation by significant inhibition of SKP2 expression in genetically engineered mouse model 171. And also, miR-378 can bind and down regulate the expression of SKP2 to control diabetic neuropathy 31. Interestingly, miRNA-181d targets 3'-UTR of *FBXL3* and stabilizing c-Myc expression and increased glucose consumption and lactate production in colorectal cancer 172. Besides, miR-4735-3p reduces the expression of FBXL3 and suppresses cell proliferation and migration in small cell lung cancer 173. The miR-1306-3p promotes cancer cell progression and metastasis by directly targeting FBXL5 through suppressing snail degradation in hepatocellular carcinoma 174**.**

FBXW7 is a tumor suppressor strongly suppressed the cancer cells proliferation, however the role of FBXW7 can be significantly inhibited by several mi-RNAs including miR-25, miR-92, miR-182, miR194, miR-223 and miR-503 in gastric, esophageal, colorectal, breast and cervical cancer cells 175-179. The expression of FBXO11 can be reduced by miR-21 with subsequent BCL6 elevation in melanoma, prostate cancer and glioma 180, 181. miR-218 suppresses FBXW8 expression and inhibits the cell proliferation in human choriocarcinoma cells 182, and miR-29c negatively regulates FBXO31 in gastric cancer cells 183. Even here we have discussed the published interaction between microRNAs and F-box proteins, however there are still many non-discovered microRNA regulating F-box proteins, and have to be elucidated.

## Small molecules and compounds are effective as inhibitors of F-box gene functions

Owing to their interactions with multiple cancer hallmark pathways, F-box proteins have been regarded as potential cancer therapeutic targets, and many small compounds have been applied to intervene F-box proteins (Table 2). SKP2 is one of the most promising targets, with various compounds showing effectiveness in inhibiting SKP2. Compound A and compound 25 interfere the binding between SKP2 and SKP1 in the SCF complex, and subsequently increases accumulation of p21, p27 and other SKP2 substrates, thereby promoting cell apoptosis 184, 185. SMIP004 can reduce the SKP2 expression in prostate cancer 186. In addition to SKP2, recognition of p27 during ubiquitination also depends on an accessory protein, Cdc kinase subunit 1 (CKS1). Compounds C1, C2, C16 and C20 have the ability to bind a structural pocket developed between SKP2 and CKS1 and block the interaction in metastatic melanoma, prostate, breast, ovarian and lung cancer 187. Besides, natural products such as curcumin, quercetin, lycopene, silibinin, EGCG and vitamin D can inhibit the expression of SKP2 in breast and prostate cancer 188-190.

β-TrCP is another promising target. GS143 disrupts interaction between phospho-IκBα and β-TrCP, thereby suppressing IκBα ubiquitylation 191. Generally, erioflorin inhibits the interaction between β-TrCP and a tumor suppressor PDCD4 in cancer 192. STG28 also modulates the expression of β-TrCP and β-catenin citing its potential chemotherapeutic efficacies 193.

In addition to SKP2 and β-TrCP, some small compounds targeting on FBXW7, FBXO3, FBXL2, and FBXL3 are under study as well. SINE KPT-185 can block nuclear export of FBXW7 and enhances nuclear retention of FBXW7 to degrade NOTCH1 194. Oridonin increases level of FBXW7 and activates GSK3 to enhance c-Myc turnover in leukaemia and lymphoma 195. BC1215 inhibits the binding of FBXO3 and FBXL2 to the target 109. Another compound BC1258 has property to block binding between FBXO3 and FBXL2 and stabilize FBXL2 to promote Aurora B degradation 44. KL001 competes for binding in the FAD pocket of CRYs and prevents FBXL3 binding in disorders including sleep disorder, cancer, cardiovascular and metabolic diseases 196 (Table 2).

Recently, chemists have developed a proteolysis-targeting chimeras (PROTACs) technology that can induce targeted protein degradation by the ubiquitin-proteasome system. PROTACs focused on drug resistance and 'undruggable' targets research. Till now less than ten E3 ubiquitin ligases have been exploited for targeted protein degradation 197. With advances in this technology, more potential F-box proteins will be discovered.

## Concluding Remarks: Future prospect and therapeutic implications of F-box gene family

Going through the updates on F-box protein mediated ubiquitination mechanisms, it is very clear that F-box proteins play key roles in vital cellular functions namely, cell cycle, genome instability, signaling pathway and apoptosis in normal as well as tumor cells. Despite an initial limited knowledge on the role of F-box proteins, multiple F-box proteins have shown either oncogenic or tumor suppressive potentials in certain types of cancer (Table 1). The clinical implications and therapeutic or prognostic importance have emerged widely and been improved in the current decade.

Although many F-box proteins have been proposed as potential cancer therapeutic targets, none of them have entered into clinical research. Large challenges remain for F-box protein targeted cancer therapy. It is obvious that most of cancer relevant pathways are controlled by multiple F-box proteins rather than only one of them, for instance, the cell cycle pathway alone is influenced by at least 10 F-box proteins (Table 1), implying the potential cooperative mechanism between different F-box proteins in regulating the cellular functions. Even the same substrates can be ubiquitinated by multiple F-box proteins, e.g., MCL1 can be degraded by β-TrCP, FBXW7 and FBXO4 (Table 1) under different contexts. Drugs simply inhibiting of one specific F-box protein are probably not enough to control cancer cell development or progression, since various compensative mechanisms exist. Additionally, it should be noted that one F-box protein (e.g., SKP2, FBXW7, etc.) generally target on multiple different substrates involved in different pathways. Before taking the F-box proteins as therapeutic targets, we must make sure the intervention will not cause unexpected consequences by influencing the multifunctional substrates. What's more, most of the ubiquitination interactions are context dependent and varied with different cancer types, the up-stream pathways or co-regulators. The corresponding therapeutic effects are also determined by their context-dependent functions. Consequently, a more comprehensive understanding about the interactome with respect to F-box proteins as well as other E3 ligases is necessary. Future drug development should pay more attention to the complicated interactome and regulatory mechanism of F-box proteins, the drugs are better to target on specific interactions between F-box proteins and substrates or co-regulators that play key functions in certain types of cancer.

Advanced state-of-art technology and evolving scientific advances across various models have poured a concrete confidence for discovery of novel mechanisms played by F-box proteins. Physical interaction mechanism study has now placed a new scope to identify the detailed structure and topology of the interacting partners or domains. Identification of upstream target genes and compounds has led us to a new therapeutic strategy for future. Before stepping at the clinical door, biological suitability and efficacy of the drugs or compounds needs more rigorous investigations across wide range of cell systems and animal models. Combination of systems and *in-depth* molecular biology will promote the illustration about the unknown mechanisms left with undetermined F-box proteins. More importantly, investigation of large pool of human clinical samples is warranted. The most challenging aspect considering the future clinical trials will be the great genetic diversity to all corners of populations in this world. Taken together, we should always keep in mind all the aforesaid findings and issues when we stick and step on this way.

## Figures and Tables

**Table 1:** Targets/substrates of F-box proteins and their biological functions

F-box protein	Localization	Role	Substrates	Biological functions
**FBXL gene family**				
SKP2	Nucleus,Cytoplasm	Oncogene	RELN	EMT 16
			CARM1	AMPK pathway 18
			AKT (K63)	AKT Pathway 167
			PDCD4	Apoptosis 34
			P21	Cell cycle 23
			P27	Cell cycle 22
			P130	Cell cycle 27
			c-Myc	Cell cycle 26
			Cyclin E	Cell cycle 25
			FOXO1	FOXO signaling pathway 33
			YAP1 (K63)	Transcription of target genes 35
FBXL2	Cytoplasm, Membrane	Potential tumor suppressor	Cyclin D	Cell cycle checkpoints^42^, ^43^
			AURKB	Cell cycle checkpoints^44^
			p85β	PI3K pathway 7
			IP3R3	PI3K pathway 11
			FOXM1	Cell proliferation 45
FBXL3	Nucleus, Cytoplasmic	Potential tumor suppressor	CRY1,CRY2	Circadian clock system 49
			c-Myc	Cell cycle progression, apoptosis and cellular transformation 50
			TLK2	Cell cycle 51
FBXL4	Cytoplasm, Mitochondrion, Nucleus	Potential tumor suppressor	KDM4A	Replication time 52
			RDL	Timing of sleep 53
FBXL5	Cytoplasm, Perinuclear region	Potential tumor suppressor	IRP1,IRP2	Iron metabolism 56 168
			p150	Genome stability^59^
			SNAIL1	EMT 60
			CITED2	HIF pathway 61
			hSSB1	DNA repair 62
FBXL7	Cytoskeleton	Potential oncogene	AURKA	Cell cycle 63
			Survivin	Apoptosis 65
FBXL12	Cytoplasm, Nucleus	Unclear	ALDH3	T cell development 70
			Ku80	Non homologues end joining double strand break repair mechanism 169
			CaMKI	Cell cycle 68
			p21	Cell cycle progression at G1 69
FBXL13	Cytoskeleton, Cytoplasm	Potential oncogene	CEP192	Centrosome duplication 72
FBXL14	Cytoplasm	Potential tumor suppressor	CDCP1	Tyrosine phosphorylation-dependent regulation of cellular events 77
			SNAIL1	EMT 75
			c-Myc	Cell cycle progression, apoptosis and cellular transformation 76
			HES1	Neuron development 78
			TWIST1	EMT 74
FBXL15	Cytoplasm	Unclear	SMURF1	BMP signaling pathway 12
FBXL17	Nucleus, Cytoplasm	Potential oncogene	SUFU	Hedgehog signal pathway^79^
			BACH1	NRF2 oxidative stress pathway 14
FBXL18	Cytoplasm, Nucleus	Potential oncogene	XPB	Transcription 81
			AKT(K63)	AKT pathway 15
FBXL19	Cytoplasm	Potential tumor suppressor	RAC3	TGFβ1-induced E-cadherin down-regulation 83
			RhoA	Cell proliferation and cytoskeleton rearrangement 84
FBXL20	Cytoplasm	Potential oncogene	E-cadherin	Wnt signaling pathway 87
			Vps34	Autophagy 86
FBXL21	Cytoplasm, Nucleus	Unclear	CRY1,CRY2	Circadian clock system 88
**FBXW gene family**				
β-TrCP	Nucleus, Cytoplasm	Generally oncogene & tumor suppressor in a few cases	β-catenin	Cell viability 90
			IκBα	NF-κB pathway 93
			CDC25A	Cell cycle 94
			REST	Spindle check points 96
			MCL1	Anti-apoptotic (a member of the Bcl-2 family) 97
			p53	p53 pathway 101
			c-Myc	Apoptosis 99
			Lipin1	Fatty acid biosynthesis 100
			MTSS1	Tumour suppression 104
			NRF2	NRF2 pathway 98
			FBXW2	β-TrCP-FBXW2-SKP2 axis 102
			ZNF281	Colorectal cancer progression 103
			DEPTOR, REDD1	Autophagy 105
FBXW7	FBXWα: Nucleoplasm, FBXWβ: Cytoplasm, FBXWγ:Nucleolus	Tumor suppressor	Cyclin E	Cell cycle 106
			mTOR	mTOR signaling pathway 110
			NOTCH1	NOTCH1 signaling 114
			c-Jun and DEK	Closed circularity of DNA, cell cycle progression 112
			MCL1	Apoptosis 111
			c-Myc	Cell proliferation 109
FBXW2	Cytoplasm	Potential tumor suppressor	SKP2	β-TrCP-FBXW2-SKP2 axis 102
			β-catenin	116
FBXW5	Cytoplasm, Nucleus	Unclear	SASS6	Centrosome duplication 118
			EPS8	Cell proliferation and motility 119
			TSC2	Tuberous sclerosis 117
FBXW8	Golgi apparatus, Cytoplasm	Unclear	MAP4K1	MAPK pathway 120
**FBXO gene family**				
FBXO1	Nucleus, Cytoplasm, Cytoskeleton	Potential tumor suppressor	RRM2	DNA replication and DNA repair synthesis 122
			CP110	Centrosome duplication; Genomic integrity 121
			NUSAP1	Chromosome assembly 123
			CDC6	Early steps of DNA replication 124
FBXO3	Nucleus, Cytoplasm	Unclear	AIRE	T cell development 128
			HIPK2,p300	Transcription 126
			SMRUF1	BMP signaling pathway 129
FBXO4	Cytoplasm	Tumor suppressor	TRF1	Cell cycle 131
			MCL1	Apoptosis 137
			FXR1	RNA binding protein and Fragile X syndrome 134
			ICAM1	Intercellular adhesion 136
			PPARγ	Adipocyte differentiation 138
			Cyclin D1	Cell cycle 130
FBXO6	Cytoplasm	Unclear	CHK1	Cisplatin sensitivity 140
			MAD2,BUBR1	Spindle checkpoint 142
			Ero1L	Apoptosis 141
FBXO7	Cytoplasm, Mitochondrion, Nucleus	Potential oncogene	cIAP1	Inhibition of apoptosis 146
FBXO10	Cytoplasm	Potential tumor suppressor	BCL1	Apoptosis 170
FBXO11	Chromosome, Nucleus	Potential tumor suppressor	HIF-1α	HIF-1αsignaling pathway 143
			BCL6	B-cells differentiation 147
			SNAIL	EMT 148
FBXO21	Cytoplasm	Unclear	EID1	Cell cycle 164
FBXO22	Cytoplasm, Nucleus, Z disc	Unclear	ACTN2, FLNC	Contractile function 165
FBXO31	Cytoplasm, Cytoskeleton	Potential tumor suppressor	SNAIL1	EMT 153
			Cyclin D1	Cell cycle 152
			MDM2	p53-mediated growth arrest 154
			MKK6	MAPK pathway 155
FBXO32	Cytoplasm, Nucleus	Unclear	KLF4	Apoptosis 156
			IκBα	NF-κB pathway 157
			CTBP1	EMT 158
FBXO38	Cytoplasm, Nucleus	Potential tumor suppressor	PD-1	Immunity of T cells 166
FBXO45	Postsynaptic cell membrane , Cell junction, Synapse	Unclear	N-Cadherin	Neuronal differentiation 163
			PAR4	Apoptosis 161
			p73	Apoptosis 162

Note: other unlisted F-box proteins have no known substrates at present. The potential roles of F-box proteins, mostly context dependent, are just based on current limited studies.

**Table 2:** Details of different molecules and compounds which target F-box proteins

Target	Compound	Identified functions
SKP2	Compound A	Disrupts SKP2-SKP1 interaction and prevents ubiquitination of p27 184
SKP2	SMIP004	Targets SKP2 and down-regulation SKP2^186^
SKP2	C1, C2, C16, C20	Binds to a pocket formed by SKP2 and CKS1 to block substrate binding 187
SKP2	Compound 25/ SZL-P1-41	Binds to SKP2 and prevents SKP2-SKP1 interaction 185
SKP2	Curcumin, Quercetin, Lycopene, Silibinin, EGCG, EGCG, Vitamin-D	Blocks SKP2 expression 188-190 .
SKP2	NSC689857 NSC681152	Blocks the SKP2-CKS1 interaction and p27 ubiquitination *in vitro* 198
β-TrCP	GS143	Interfere interaction between phospho-IkBα and β-TrCP, suppress IkBα ubiquitylation 191
β-TrCP	Erioflorin	Blocks the interaction of β-TrCP to PDCD4 192
β-TrCP	STG28	Modulates the expression of β-TrCP and β-catenin 193
FBXW7	SINE KPT-185	Inhibits transport of FBXW7, increases nuclear FBXW7 level and degrades NOTCH1 194
FBXW7	Oridonin	Increases FBXW7 level, activates GSK3 and facilitates c-Myc degradation 195
FBXW7	Genistein	Down-regulates miR-223 level and elevates its target FBXW7 level 199
FBXW7	SCF-12	Interferes substrate binding pocket and impede recognition of phosphodegron on substrates 6
FBXL2	BC-1215	Inhibits FBXO3 and FBXL2 binding 44
FBXL2	BC-1258	Inhibits binding FBXO3 and FBXL2; stabilizes FBXL2 and promotes AURKB Degradation 44
FBXL3	KL001	Competes for binding in the FAD pocket of CRYs and prevents FBXL3 binding 200
FBXO3	BC1215	Inhibits the substrate binding to FBXO3 44
